# Microcystins Presence in Mussels* (M. galloprovincialis)* and Water of Two Productive Mediterranean's Lagoons (Sardinia, Italy)

**DOI:** 10.1155/2017/3769245

**Published:** 2017-11-21

**Authors:** Elena Baralla, Maria Vittoria Varoni, Tiziana Sedda, Valeria Pasciu, Antonello Floris, Maria Piera Demontis

**Affiliations:** Department of Veterinary Medicine, University of Sassari, Via Vienna 2, 07100 Sassari, Italy

## Abstract

Microcystins (MCs) are hepatotoxins harmful for animal and human health. The most toxic type between them is MC-LR whose presence has been investigated in different reservoirs all around the world. In this work microcystins were monitored in spring and summer in water and mussels* (Mytilus galloprovincialis)* of two Sardinia lagoons: Cabras and Calich lagoons. A Solid Phase Extraction method was developed to clean and concentrate samples before the Enzyme Linked Immunosorbent Assay (ELISA) and the following Mass Spectrometry detection. MCs presence was detected using the screening ELISA test in both lagoons. MCs peak was revealed in July for water and mussels belonging to Cabras lagoon (0.75 ± 0.07 ng/L in water and 0.12 ± 0.04 ng/g ww in mussels). In water of Calich lagoon there was a constant trend in the concentration of MCs during the considered months, while there was a MCs peak in July (0.6 ± 0.5 ng/g ww) in mussels. The following LC-MS/MS analysis did not reveal MC-LR presence in all analyzed samples. These results can be useful to enrich knowledge on public health and consumer's safeguard.

## 1. Introduction

Cyanobacteria (blue-green algae) are a group of more than 2000 species of prokaryotic organisms present in almost every terrestrial and aquatic habitat [1, 2]. Approximately 40 genera of Cyanobacteria produce secondary metabolites called cyanotoxins [3]. These are different in chemical structure and toxicity and they can be classified into four families according to the organ on which they act: neurotoxins, hepatotoxins, cytotoxins, and dermatotoxins [4].

Microcystins (MCs) are a group of over 90 hepatotoxins produced from multiple genera of Cyanobacteria* (Microcystis, Anabaena, Oscillatoria, Planktothrix, Chroococcus*, and* Nostoc)*. They are cyclic heptapeptides with the general structure cyclo(-d-Ala-l-X-erythro-b-methyl-d-isoAspl-Y-Adda-d-isoGlu-N-methyldehydro-Ala) and contain an unusual amino acid (2S,3S,8S,9S)-3-amino-9-methoxy-2,6,8-trimethyl-10-phenyldeca-4,6-dienoic acid (Adda) that is essential for their biological activity [5]. Main structural variations in MCs are the L-amino acid residues X and Y, so that they are named using the abbreviation for the amino acids substituted. Between MCs, microcystin-LR (MC-LR) is the most common and toxic [2, 6, 7]. It contains leucine (L) and arginine (R) as variable amino acids [8]. MCs are irreversible inhibitors of serine/threonine protein phosphatases (PP1 and PP2A), an effect described in zooplankton, amphibians, fishes, and mammals [9]. Liver is the most affected organ in humans but the exposure to this toxin is likely to affect also other organs such as kidney and colon as evidenced by* in vivo* and* in vitro* studies [10]. MCs can cause oxidative stress by increasing the formation of reactive oxygen species (ROS) and mitochondrial changes, two mechanisms involved in tumor initiation [11]. Given these premises, the International Agency for Research on Cancer (IARC) considered MC-LR as “possibly carcinogenic to humans” (group 2B) and the World Health Organization (WHO) established a provisional tolerable daily intake (TDI) of 0.04 *μ*g of MC-LR bodyweight/day and a provisional guidelines value for MC-LR of 1 *μ*g/mL in drinking water [12]. In June 2015, EPA has developed two health advisory values—a ten-day health advisory (HA) of 0.3 *μ*g/L based on exposure to infants over the first year of life and a ten-day HA of 1.6 *μ*g/L based on exposure to adults, over 21 years of age. It is therefore evident that exposure to MCs constitutes a high health risk to both animals and humans considering also the consumption of contaminated animals and seafood that can accumulate MC-LR and that are important food sources [3, 8].

Although accumulation of microcystin is known to occur, no widely accepted guidelines have been established for microcystin concentrations in fish tissue and potential exposure through fish consumption [13]. Mussels are known as biological markers of pollution and several studies have shown the capacity of mussels to accumulate MCs [14–16]. MCs presence was investigated in some Sardinian reservoirs [17, 18] and, to our knowledge, only our previous study investigated MCs presence in clams of two Sardinian lagoons [19].

The aim of this work was to investigate MCs presence in water and mussels* (M. galloprovincialis)* belonging to two important coastal lagoons of Sardinia, known for their productive and touristic relevance. Results obtained will enrich knowledge on public health and consumer's safeguard according to the relevant legislation.

## 2. Materials and Methods

Samples of water and mussels* (M. galloprovincialis)*, belonging to two important coastal lagoons of Sardinia (Cabras and Calich), have been analyzed. Cabras lagoon is the largest and one of the most productive Sardinian lagoons and during the twentieth century its connection with the sea has been strongly modified by human intervention. Calich lagoon is a small Sardinian lagoon located in an area of strong touristic impact. Mussels and water were collected in spring and summer 2015 and analyzed with two different analytical techniques: a first preliminary screening Enzyme Linked Immunosorbent Assay (ELISA) to evaluate MCs presence in the samples and a successive liquid chromatography-tandem mass spectrometer (LC-MS/MS) analysis to reveal MC-LR.

### 2.1. Chemicals and Reagents

MC-LR standard was obtained from 3V Chimica S.r.l. (Roma, Italy). Deionised and distilled water was purified through a MilliQ water system (Millipore, Bedford, MA, USA). MS grade solvents were purchased from Sigma Aldrich (Milan, Italy). CHROMABOND C_18_ SPE columns (500 mg/6 mL) were purchased from Exacta Optech Labcenter (S. Prospero-MO-Italy) and mounted on a Vacuum Manifold Set (Phenomenex, Castelmaggiore, Italy). The EnviroGard® ELISA Kit was purchased from ECOTOX (Cornaredo-Mi, Italy). MC-LR stock solution (0.1 mg/mL) was prepared in methanol and used to prepare working solutions by appropriate dilution.

### 2.2. Sites Description

The two coastal lagoons are located in the western coast of Sardinia.

Cabras lagoon is connected with the Oristano Gulf through the Scolmatore (=spillway) canal and with some rivers as Rio Mare e Foghe. Calich lagoon is situated on the north western Mediterranean Sea and in the Porto Conte Regional Natural Park. It is connected with the Alghero Gulf through a natural channel and with three different rivers. The two lagoons suffer of high eutrophication due to urban, agricultural, and industrial activities [14–16]. Water temperature in both lagoons follows a seasonal trend with highest values between July and August.

**Figure 1:**
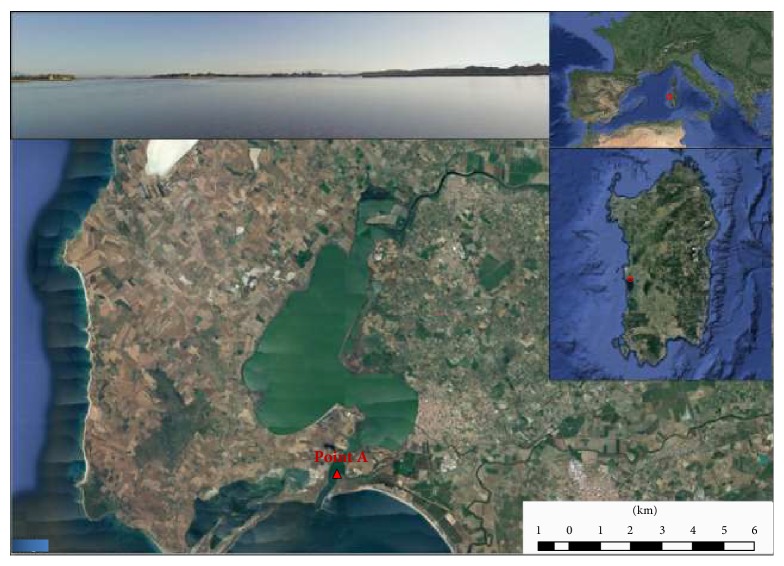
Map of Cabras lagoon with the geographic coordinates of sampling site.

**Figure 2:**
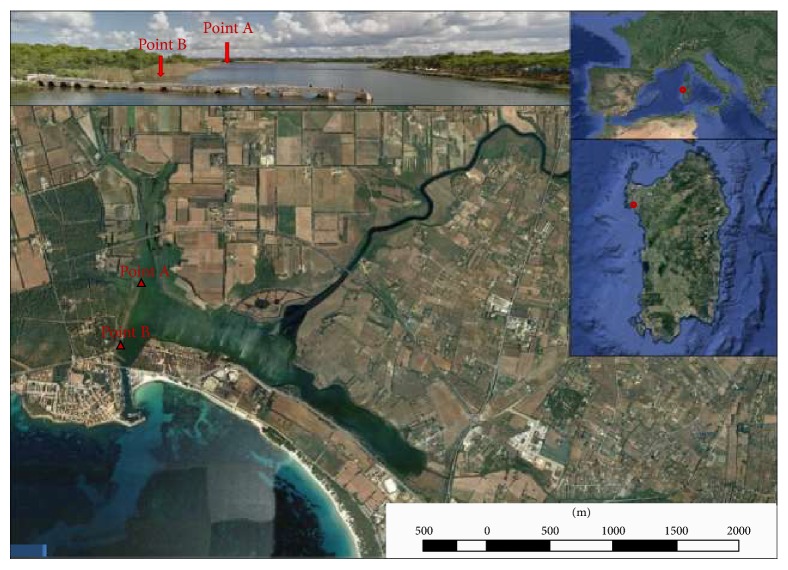
Map of Calich lagoon with the geographic coordinates of sampling sites: water was collected in both sites, while mussels were, in part, harvested in the site nearer to the sea and, in part, bought from local fishermen.

Geographic coordinates of sampling sites of mussels and water are shown in Figures 1 and 2 for Cabras and Calich, respectively.

### 2.3. Sample Collection and Preparation

Samples were prepared according to the procedures indicated in the “Bulletin of Analytical Methods of the National Health Institute” [20].

Water was collected using amber glass bottles and stored at 4°C till the subsequent filtration step within 48 hours. 1 L of water was filtered using Whatman glass microfiber filters GF/F diameter 47 mm (Sigma Aldrich, Milano, Italy). The filtrate was concentrated using solid phase extraction (SPE) columns Chromabond C18 as described next.

Mussels collected in the two lagoons were homogenized and frozen at −20°C.

After thawing, 5 g of homogenate was transferred into a 50 mL falcon and added to 10 mL of methanol: formic acid 0,2% 9 : 1 v/v. After vortexing, the mixture was extracted in an ultrasonic bath for 1 hour at room temperature before being centrifuged at 1400 g for 30 minutes. The supernatant was then evaporated to a minimum volume under nitrogen stream and reconstituted with a solution of formic acid 0.2% in MilliQ water. Both water and mussels samples were successively extracted using SPE before the ELISA and LC-MS/MS analysis.

### 2.4. Microcystin SPE Extraction from Water and Mussels

Sample extraction, for both matrices, was performed as already described by Mekebri et al. (2009) [21] with minor modifications. In detail, water filtrate and mussels treated as described above were cleaned using SPE C_18_ columns (containing 500 mg of adsorbing phase) previously activated and conditioned with 3 mL of methanol and 6 mL of MilliQ water. After sample loading, the column was washed with 3 mL of MilliQ water and 3 mL of a mixture containing water and methanol 80 : 20. The column was dried under an air stream for 15 min and then 5 mL of 0.1% formic acid in methanol followed by 5 mL of 0.1% formic acid in a mixture of methanol and water 75 : 25 was used to elute the analyte. At the end of this procedure, solvents were removed by evaporation under nitrogen stream, and the residue was reconstituted with two different solvents depending on the technique used for the analysis. For ELISA test, samples were reconstituted with 500 *μ*L of acidified water, while, for LC-MS/MS, the reconstitution solvent was 500 *μ*L of mobile phase. The difference between the two solvents used can be explained considering that methanol can interfere with the ELISA sensibility. This SPE procedure permitted not only cleaning samples in order to eliminate interferences for the following analysis but also concentrating the analyte.

### 2.5. Microcystin Analysis

#### 2.5.1. ELISA Test

An EnviroGard Microcystin Plate Kit was used for the MCs assay with the ELISA technique. It is based on a direct competitive ELISA for quantitative detection of MCs and nodularins according to the manufacturer's instructions and on the polyclonal antibodies of Chu et al. (1989) [22]. In detail, 100 *μ*L of negative control, calibrator, and sample was added to their respective wells before being incubated for 30 min at room temperature. After that, to each well, 100 *μ*L of microcystins-enzyme conjugate was added and mixed. After another incubation of 30 minutes, a wash step with deionised water was repeated four times. Then, 100 *μ*L of substrate was added to each well before another incubation of 30 min at room temperature. Finally 100 *μ*L of stop solution was added to each well. The obtained color and its related absorbance, where toxin concentration is inversely proportional to color development, were read at 450 nm within 30 min. MCs values were calculated using a standard curve (0.1–0.6 ng/mL) and normalized for tissue wet weight (ww) for mussels.

#### 2.5.2. LC-MS/MS Analysis

An HPLC ProStar™ 300 (Varian, Palo Alto, CA, USA) instrument was used. The chromatographic process was carried out on a Phenomenex Luna C_18_ column (5 *μ*m, 100 × 2.0 mm, Phenomenex, Torrance, CA, USA) fitted with a C_18_ security guard cartridge (4 × 2 mm ID). A sample volume of 5 *μ*L was injected into the LC-MS/MS system. A linear gradient with 0.2% formic acid in water (A) and 0.2% formic acid in acetonitrile (B) was performed. A percentage of 20% of B was maintained for 1 min and then increased to 80% in 2 min and remained constant for 2.3 min. Gradually, in 6 sec, solvent B was decreased from 80% to 20% and it remained constant for 2.54 min for a total runtime of 8.30 min, with a flow rate of 0.2 mL/min. MS detection was performed on a Varian 310-MS triple quadrupole mass spectrometer (Varian, Palo Alto, CA, USA) equipped with an electrospray ionization (ESI) interface in positive ion mode. A direct infusion of standard solution (500 ng/mL) was made with mobile phase at a flow of 400 *μ*L/min for the optimization of detection conditions. The ESI source conditions were as follows: capillary voltage, 80 V; drying gas temperature, 200°C; nebulizer gas pressure, 50 psi (both nebulizer and drying gas were high-purity nitrogen); detector voltage, 1900 V. Argon was used for the collision with a pressure of 2 mTorr. Collision energies (CE) were optimized for each product ion, for a maximum detection. The multiple reaction monitoring (MRM) mode was used. For the analysis of MC-LR two transitions were chosen, with the most abundant between them (996* m/z *→ 213* m/z* CE = 45 eV) used for the quantitation and the other one (996* m/z *→ 135* m/z* CE = 50 eV) to confirm the analyte identity. A six-level calibration curve, from 5 ng/mL to 500 ng/mL of certified MC-LR, was prepared in methanol-water 90 : 10 v/v using the external standard method. The limit of determination (LOD) was obtained using the signal to noise criteria of 3, while the limit of quantitation (LOQ) was calculated using the signal to noise criteria of 10 and was 10 ng/mL and 20 ng/mL, respectively.

### 2.6. Statistical Analysis

All values are expressed as mean ± SE. Results were analyzed by one-way analysis of variance (ANOVA) and Student's *t*-test. Statistical significance was accepted when *p* < 0.05. Statistical calculations were performed using the software program Statgraphics (Statpoint Technologies, Warrenton, VA).

## 3. Results and Discussion

A deep study of the extraction and preparation method for the analysis of MCs from tissues and water has been carried out in order to identify the most suitable one.

### 3.1. ELISA Test

**Figure 3:**
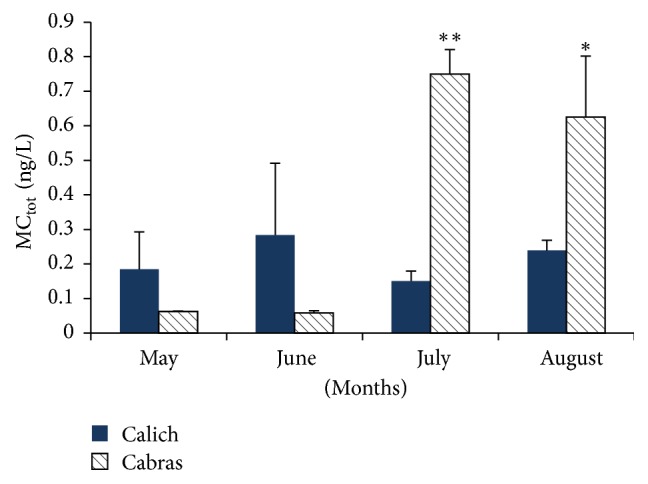
ELISA results of MCs levels in water in Calich and Cabras lagoons. Data are expressed as mean ± SE. Asterisks indicate statistical differences in respect to May and June. ^*∗*^*p* < 0.05; ^*∗∗*^*p* < 0.001.

ELISA assay was performed on water and mussels samples collected in spring and summer from Cabras and Calich lagoons. In particular we focused on the months of May, June, July, and August considering that in these months there is an increase of the water temperature of these lagoons [15, 23]. Moreover, during summer, high environmental temperatures are connected with calm wind and drought, all parameters that cause cyanotoxins release [24, 25]. Elisa test revealed the presence of MCs in water during spring and summer with a peak in July (0.75 ± 0.07 ng/L; *p* < 0.001) and August (0.63 ± 0.18 ng/L; *p* < 0.05) for Cabras lagoon, while there was a constant trend between May, June, July, and August for Calich lagoon (mean concentration: 0.20 ± 0.06 ng/L). These results are shown in Figure 3.

Although MCs presence was determined in the described months, the found concentrations were very low and this is a comfortable result for water quality.

**Figure 4:**
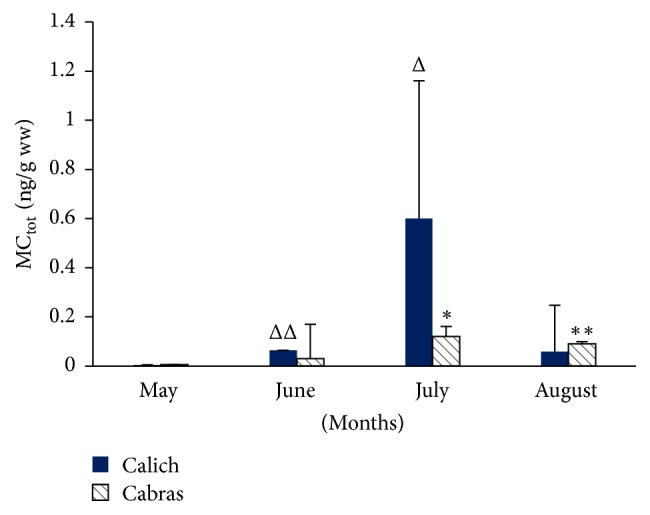
ELISA results of MCs levels in mussels in Calich and Cabras lagoons. Data are expressed as mean ± SE. Asterisks indicate statistical differences in respect to May and June for Cabras (^*∗*^*p* < 0.05; ^*∗∗*^*p* < 0.001) and triangles indicate statistical differences in respect to May and August for Calich (Δ < 0.05; ΔΔ < 0.001).

As regards mussels, MCs was revealed during July (0.12 ± 0.04 ng/g ww; *p* < 0.05) and August (0.09 ± 0.01 ng/g ww; *p* < 0.01) in Cabras lagoon and during June (0.05 ± 0.00 ng/g ww; *p* < 0.05) and July (0.60 ± 0.50 ng/g ww; *p* < 0.01) in Calich lagoon. These results are shown in Figure 4.

The low MCs concentrations found in mussels suggest the healthiness of these products.

The ELISA kit used, as all the available commercial kits, is not able to differentiate between the different MCs and congeners. This is because they recognize the ADDA moiety that is present in all MCs and congeners. However ELISA tests are usually used as screening methods because they are fast and easy to carry out. Results obtained through this technique need to be confirmed by more specific and sensible techniques such as the LC-MS/MS.

### 3.2. LC-MS/MS

**Figure 5:**
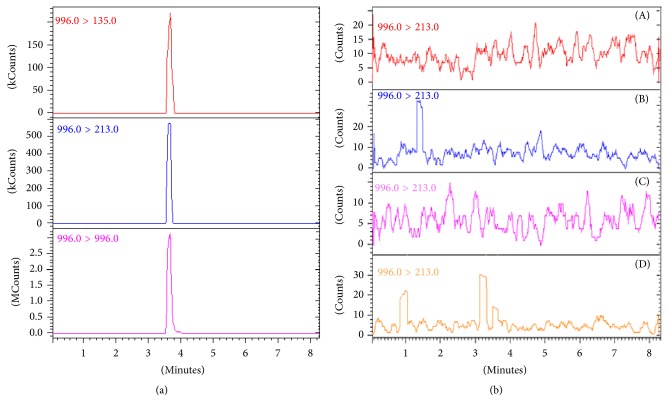
(a) Multiple reaction monitoring chromatogram of a standard solution of MC-LR (500 ng/mL). (b) Chromatograms of water and mussels harvested in Calich lagoon ((A), (B), resp.) and Cabras lagoon ((C), (D), resp.) in July.

Water and mussels samples that gave positive results in ELISA were successively analyzed in LC-MS/MS. This analysis was specific for the identification and quantitation of MC-LR, given that it is the most toxic between MCs. The chromatogram of a standard solution containing 500 ng/mL of MC-LR is shown in Figure 5(a).

Water and mussels analyzed did not contain MC-LR. A concentration step was performed before all the analyses in order to achieve lower limit of detection and quantitation (dilution factors were 2000 and 20 for water and mussels, resp.). For this reason we can assume that MC-LR is not present at concentrations higher than 0.01 ng/mL and 2 ng/g ww in water and mussels, respectively. An example of a chromatogram obtained analyzing water and mussels is shown in Figure 5(b).

Given that MC-LR was not found above the limit of quantitation of this confirmatory technique, we can assume that MCs concentrations found through the ELISA technique were attributable to other microcystins and congeners. This can be considered a positive result given that MC-LR is known to be one of the most abundant and toxic MCs variants [26, 27]. This study focused on the analysis of samples harvested during spring and summer, given the environmental factors, as stagnant water conditions and higher temperatures, favorable to cyanotoxins spread [28, 29].

Pulina et al. [30] reported several anoxic crises during summers in Cabras lagoon and, between them, the strongest one occurred in summer 1999. It is possible that these anoxic crises can cause in the following months the mortality of lagoon species [31, 32]. Furthermore, the presence of some Cyanobacteria in the lagoon decreases enormously only after strong rainfall in autumn and winter when the large input of freshwater caused a decrease in salinity [32]. Further studies will be carried out in order to monitor MCs presence in the months preceding and following the months studied in this work.

## 4. Conclusion

The absence of MC-LR at concentrations higher than 0.01 ng/mL in water and 2 ng/g ww in mussels of Cabras and Calich lagoons constitutes significant information to the healthiness of water and products of these lagoons. Both lagoons are in fact actually used for fishing and aquaculture. The developed method permitted to reveal MC-LR at a very low concentration, markedly under the limits indicated by WHO and EPA. Results obtained in this work are very interesting considering the economic, touristic, and environmental points of view. These can be useful to enrich knowledge on public health and consumer's safeguard according to the relevant legislation.
